# Chromosome Aberrations in Cells Infected with Bovine Papillomavirus: Comparing Cutaneous Papilloma, Esophagus Papilloma, and Urinary Bladder Lesion Cells

**DOI:** 10.1155/2013/910849

**Published:** 2013-11-05

**Authors:** S. R. C. Campos, T. C. Melo, S. Assaf, R. P. Araldi, J. Mazzuchelli-de-Souza, M. P. Sircili, R. F. Carvalho, F. Roperto, W. Beçak, R. C. Stocco

**Affiliations:** ^1^Laboratório de Genética, Instituto Butantan, Avenida Vital Brasil, 1500, Butantã, 05503-900 São Paulo, SP, Brazil; ^2^Programa de Pós-graduação em Biologia Estrutural e Funcional, Universidade Federal de São Paulo, Rua Botucatu, 740, 04023-900 São Paulo, SP, Brazil; ^3^Programa de Pós-graduação Interunidades em Biotecnologia, Instituto de Ciências Biomédicas, Universidade de São Paulo, Avenida Prof. Lineu Prestes, 2415 Edifício ICB-III-Cidade Universitária, 05508-900 São Paulo, SP, Brazil; ^4^Department of Biology, Naples University Federico II, Via Mezzocannone 16, 80134 Naples, Italy; ^5^Departamento de Biologia, Universidade Federal da Integração Latino-Americana (UNILA), Avenida Tancredo Neves, 6731 bloco 4, 85867-970 Foz do Iguaçú, PR, Brazil

## Abstract

The majority of malignant cells present genetic instability with chromosome number changes plus segmental defects: these changes involve intact chromosomes and breakage-induced alterations. Some pathways of chromosomal instability have been proposed as random breakage, telomere fusion, and centromere fission. Chromosome alterations in tumor cells have been described in animal models and *in vitro *experiments. One important question is about possible discrepancies between animal models, *in vitro *studies, and the real events in cancer cells *in vivo*. Papillomaviruses are relevant agents in oncogenic processes related to action on host genome. Recently, many reports have discussed the presence of virus DNA in peripheral blood, in humans and in animals infected by papillomaviruses. The meaning of this event is of controversy: possible product of apoptosis occurring in cancer cells, metastasized cancer cells, or active DNA sequences circulating in bloodstream. This study compares chromosome aberrations detected in bovine cells, in peripheral blood cells, and in BPV lesion cells: the literature is poor in this type of study. Comparing chromosome aberrations described in the different cells, a common mechanism in their origin, can be suggested. Furthermore blood cells can be evaluated as an effective way of virus transmission.

## 1. Introduction

The papillomaviruses (PVs) are viruses that require the environment of a differentiating epithelium for their replication cycle [1]. PVs infect mammals, including man, and are related to development of benign lesions that can progress to cancer [2]. Uterine cervical cancer, the second most frequently occurring cancer in women worldwide, is causal related to human papillomavirus infection (HPV) [3, 4].

The Papillomavirus genome comprises three regions: LCR, “long control region” responsible for genome transcription control, L region, encoding the capsid major and minor proteins (L1 and L2, resp.), presenting late transcription in virus replication cycle, and E region, with early transcription in virus cycle, which codifies the proteins related to carcinogenic action [5, 6]. The virus transmission is recognized as occurring through direct contact: the abrasion of the skin or sexual intercourse leads to PV infection [7]. PV oncoproteins are the source for the alterations related to carcinogenesis: they interfere with the host cell cycle control, through interactions with specific proteins, as p53, p RB, p21, and p27 [8]. As examples of viral oncoprotein actions, E6 induces accelerated degradation of p53 [9]. E7 binds and degrades p RB and interacts with p21 and p27 [10]. 

Papillomaviruses maintain their genomes in episomal condition, linked to host cell chromosomes during cell division and being distributed in dividing cells [11]. The papillomavirus E2 protein simultaneously associates the host chromatin and the viral genome during mitosis [12]. The virus in its episomal form is found in benign lesions and E2 also acts in the integration of virus sequences in host chromatin of neoplasic cells. These cells are tightly associated with the expression of high-risk human papillomavirus (HPV) oncogenes E6 and E7 and exhibit chromosomal instability [13]. 

The virus is specific to epithelium, but, recently, many reports have described virus DNA sequences in blood stream [14–21]. The exact source of these sequences is not clear: circulating virus DNA has been discussed as product of lyses of circulating cancer cells or micrometastasis from the tumor [18]. Bovine Papillomavirus (BPV) DNA was simultaneously detected in different tissues of the same animals, including blood, thus suggesting a blood stream virus spread [17, 22–24].

It has been speculated that blood mononuclear cells, that migrate to sites of tissue inflammation could act as a source of virus (BPV, e.g.,) in the infection of epithelial cells and could become involved in tumor development independently or jointly with several biological and/or chemical cofactors [14, 20]. 

The seminar questions are as follows: could these sequences represent virus replicate status in the bloodstream, in particular in the lymphocytes, acting as a reservoir of viral infection? Can the viruses present oncogene expression in other tissues? 

Papillomavirus DNA sequences have been identified in blood stream of specifically bovines in recent reports [16–19, 25, 26]. The studies analyzed whole blood, plasma, isolated lymphocytes, and short-term peripheral lymphocyte cultures: the BPV DNA was detected in all systems [15, 17, 18, 22, 23]. Besides, BPV DNA was detected also in semen, uterus, urine, milk, and seminal fluid [17]. Furthermore, the cytogenetic analysis performed in short-term peripheral lymphocyte cultures revealed significant increase of chromosome aberrations in samples from infected animals [15–18]. The mechanisms of virus action on host chromatin became important issue of study. 

In this study, we analyzed the levels and the types of chromosome aberrations verified in cultured cells obtained in samples collected from peripheral blood, cutaneous papilloma, esophagus papilloma, and urinary bladder cells of animal presenting enzootic hematuria. The samples were collected from a bovine infected with bovine papillomavirus and normal controls. Short-term lymphocyte cultures were performed with the blood samples and the lesion fragments were used to obtain primary cell cultures. 

## 2. Material and Methods

### 2.1. Ethical Statements

The protocols used in this study were approved by the Ethical Committee in Research of the *Universidade Federal de São Paulo* (number 0835.07) assigned by the president of this committee. All efforts are made to minimize any suffering in the animals. 

### 2.2. Selection of Animals

Nine animals (*Bos taurus*) were selected for this study: 2 males and 7 females, comparable age. The animals were submitted to clinical evaluation performed by a veterinary. All procedures followed the ethical principles. The animal group included 2 animals clinically normal, 3 presented severe cutaneous papillomatosis, 2 had esophagus papillomas, and 2 were affected by enzootic hematuria, presenting papilloma like lesion in the bladder. 

### 2.3. Samples

Blood samples were collected in two sterile syringes with heparin or EDTA, respectively, for cultures and DNA extraction. Tissue fragments were obtained by surgical procedures performed by a veterinary with local anesthesia. 

### 2.4. Histological Analysis

For histological analysis, fragments of the samples obtained from the solid lesions were fixed in 10% neutral buffered formalin and embedded in paraffin wax: 5 *μ*m ticked sections were stained with hematoxylin/eosin. 

### 2.5. Short-Term Lymphocyte Cultures

Eighteen drops of blood were incubated in RPMI medium supplied with 10% fetal serum and 2% phytohemagglutinin during 72 hours at 37°C. 

### 2.6. Primary Cell Cultures

The tissue fragments were washed in PBS, submitted to collagenase 0.01%, 30 minutes, 37°C, and incubated in Dulbecco MEM supplied with 10% fetal serum in CO_2_ atmosphere at 37°C. 

### 2.7. Cytogenetic Preparations

Colchicine 16 *μ*g/mL, 0.1 mL was added to culture cells for 1 hour at 37°C. The cells were submitted to hypotonic solution, KCl 0.075 M, at 37°C, 10 minutes. After that, the cells were fixed in three baths with 3 : 1, methanol : acetic acid. The slides were stained in Giemsa 3%, 5 minutes.

### 2.8. Cytogenetic Analysis

The cells of each culture type were analyzed and recorded in Photomicroscopy Axiophot Zeiss. Each type of chromosome aberration was recorded, according to Melo et al. [19]. 

### 2.9. Statistical Analysis

The cytogenetic data were statistically analyzed using Students *t*-test [26].

### 2.10. Virus Identification

#### 2.10.1. Tissue and Blood DNA Extraction

The DNA extraction was performed using the Cell & Tissue Kit Illustra GenomicPrep Mini Spin (GE Healthcare, Buckinghamshire, UK), and the extracted DNA was kept at −20°C. The quality of obtained DNA samples was verified using the polymerase chain reaction (PCR) technique, by means of the amplification of a bovine *β*-globin gene fragment, (450 bp), with specific primers (*F: 5*′*-aacctctttgttcacaac cag-3*′* and R: 5*′*-cag atgcttaacccactgagc-3*′), according to Yaguiu et al. [24].

### 2.11. Viral Identification

Viral molecular identification was performed using the degenerate primers FAP59 (*forward, *5′-TAACWGTIGGICAYCCWTATT-3′) and FAP64 (*reverse, *5′-CCWATATCWVHCATITCICCATC-3′), which promote L1 gene amplification, resulting in a fragment of 478 bp, Ogawa et al., 2004 [27]. In detail, the amplification reactions were performed in a PTC-100TM (MJ Research, Inc.) thermo cycler, with PCR Master Mix (Promega, Madison, USA), under the following conditions: 10 min at 94°C, followed by 45 cycles of 1 min and 30 s at 94°C, 2 min at 52°C and 1 min and 30 s at 72°C, and a final extension step of 5 min at 72°C, for primer FAP59/FAP64. For specific BPV1, 2, and 4: *BPV-1 (5-ggagcgcctgctaac tat agg a-3*′*/5*′*-atctgttgtttgggtggtgac-3*′*), BPV-2 (5*′*-gttatacca ccc aaagaagaccct-3*′*/5*′*-ctggttgcaacagctctctttctc-3*′*), and BPV-4 (5*′*-gctgaccttccagtctta at—3*′*/5*′*-cag tttcaatctcctcttca-3*′), in detail, the amplification reactions were performed in a PTC-100TM (MJ Research, Inc.) thermo cycler, with PCR Master Mix (Promega, Madison, USA), under the following conditions: 3 min at 94°C, followed by 35 cycles of 50 s at 94°C, 1 min at 60°C and 1 min at 72°C, and a final extension step of 5 min at 72°C. The PCR products were analyzed in 2% agarose gel electrophoresis stained with GelRed in TAE buffer, visualized under UV light. 

## 3. Results and Discussion

### 3.1. Results

**Figure 1:**
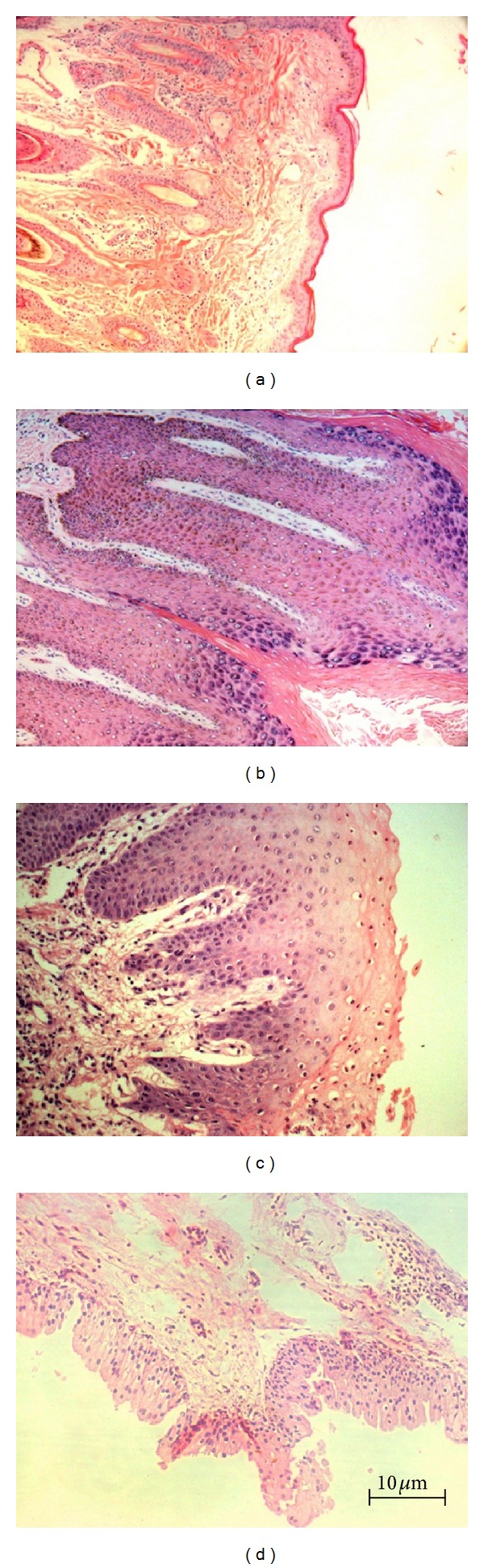
Histological sections: (a) normal skin fragment, (b) cutaneous papilloma fragment, (c) esophagus papilloma fragment, and (d) fragment of papilloma collected from an urinary bladder of an animal affected by enzootic hematuria (HE) (10x).

The histological analysis confirmed the identification of the fragments collected for the studies (Figure 1). Specifically, a segment of a normal skin obtained by surgical procedure from a normal bovine was selected as control. By similar procedures, biopsies were obtained from cutaneous papilloma, esophagus papilloma, and papilloma from urinary bladder of an animal affected with enzootic hematuria. 

**Figure 2:**
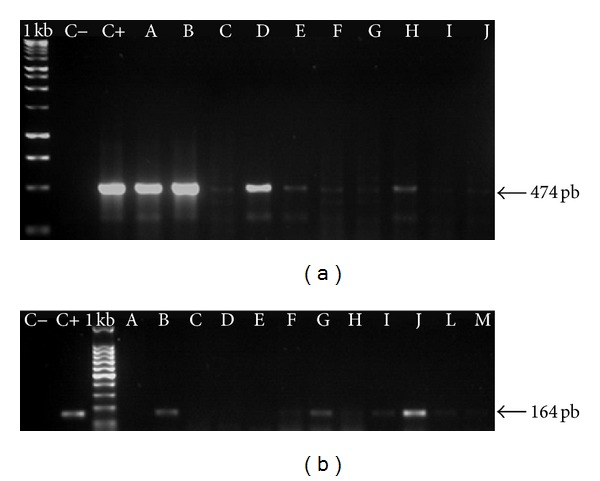
Example of BPV DNA sequences detected in cutaneous lesions and in respective primary culture cells: the samples were collected from one of the animals. I primers FAP59/64, II BPV2 specific primers. C− negative control, C+ positive control, A-B PCR with DNA from papilloma lesion, C–E primary cell culture using fragments from BPV positive lesion in passage 1, F–H primary cell culture using fragments from BPV positive lesion in passage 2, I-J primary cell culture using fragments from BPV positive lesion in passage 3, L primary cell culture using fragments from BPV positive lesion in passage 4, and M primary cell culture using fragments from BPV positive lesion in passage 5. It is important to pay attention in the different amplicons, suggesting possible different virus load.

**Figure 3:**
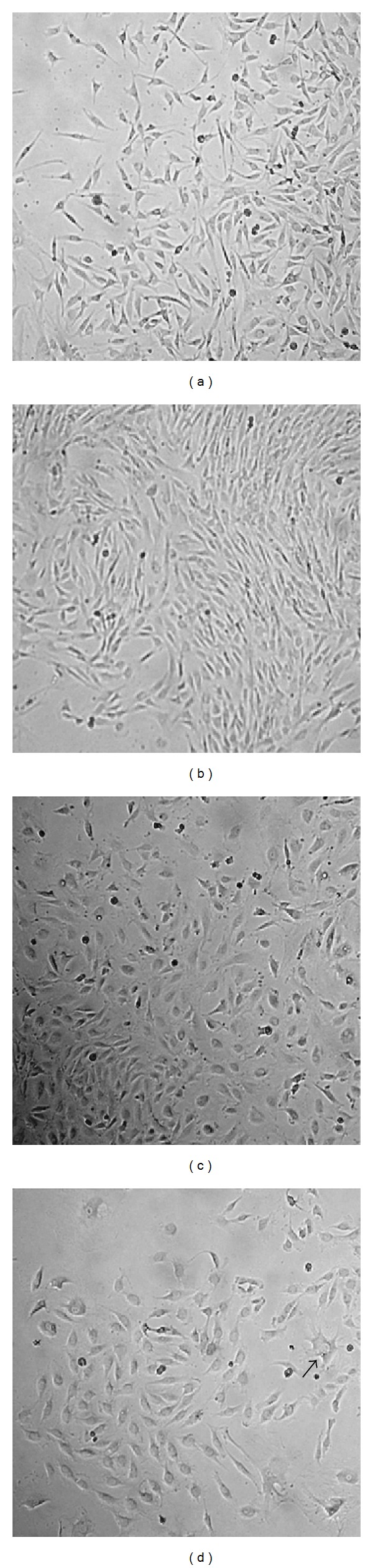
Primary cell cultures: (a) normal skin; (b) cutaneous papilloma; (c) esophagus papilloma; (d) papilloma of urinary bladder from animal affected with enzootic hematuria (10x). The arrow indicates a cell with a different morphology.

The PCR technique using primers FAP 59/64 amplified a DNA fragment 474 bp long indicating virus sequence presence in the biopsies collected from papilloma lesions. The specific primers allowed the identification of BPV1 (301 bp) and BPV2 (164 bp) (Figure 2). Only one animal presented BPV4 (170 bp) in cutaneous papilloma. Also, the same virus sequences were detected in the obtained primary cell cultures and in their first five passages (Figures 2 and 3). The same PCR procedures with the same primers were used to investigate the presence of virus sequences in blood samples, but it was not possible to detect any virus sequence in any of the blood samples, normal or affected animals. 

The Figure 3 presents cells in primary cultures. The cell morphology did not differ among the different lines. 

For cytogenetic analysis, four groups of animals were fixed according to the clinical features: C: control (not affected and without detection of virus DNA in culture cells), *Group 1*: cutaneous papilloma; *Group 2*: esophagus papilloma; *Group 3*: enzootic hematuria. A total of 1068 cells were evaluated: 501 derived from short-term lymphocyte cultures and 567 obtained from biopsy primary cell cultures (Tables 1 and 2). 

The results observed in culture blood cells make it possible to verify that the affected animals showed higher levels of chromosome aberrations compared to not affected animals. As the PCR procedures were unable to detect BPV sequences in blood cells, the data were analyzed as not affected animals compared to affected bovines. However, the PCR technique showed BPV DNA sequences in the biopsies collected from the affected animals such as the fragments from not affected animals that were negative for BPV in PCR procedures. So, the data were analyzed comparing BPV positive and BPV negative biopsies and respective primary culture cells. It is important to emphasize that the BPV negative biopsies were obtained from the not affected animals and so these animals were our controls. A specific analysis was performed considering the different chromosome aberration types comparing the previous groups (C, 1, 2, and 3). In blood cells, comparing to control, the aberration types presenting significant higher levels in cells from affected animals were addition/deletion, chromatid breaks, acentric fragments, and centromere associations (Table 2). 

**Figure 4:**
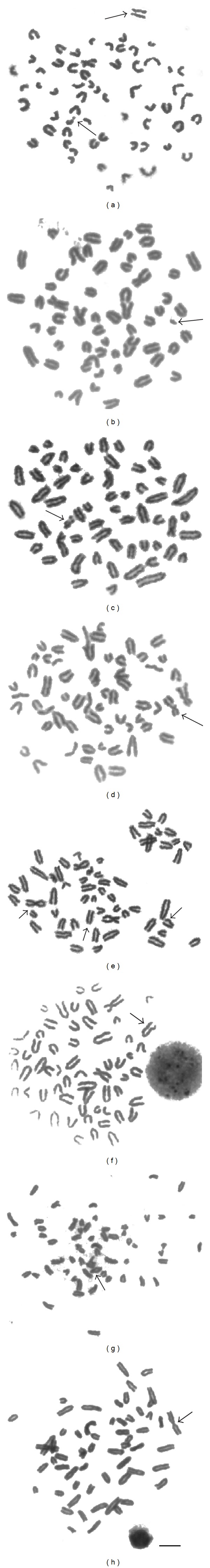
Chromosome aberrations in primary culture cells ((a), (c), (e), and (g)) and peripheral lymphocytes ((b), (d), (f), and (h)): acentric fragments ((a), (b)); chromosome break (c); chromatid break (d); chromosome rearrangement with addition/deletion ((e), (f)); telomeric association (g), centromeric association ((e), (h)); early chromatid separation (thin arrow in (a), (e)).

Considering the primary culture cells, the more frequent chromosome aberrations were addition/deletion, chromatid break, chromosome breaks, acentric fragments, centromere association, and association of telomeres (Table 2 and Figure 4). It was possible to verify that the cells obtained from the BPV infected animals presented significant higher levels of chromosome aberrations. 

### 3.2. Discussion

As far it was possible to verify, this is the first report describing chromosome aberrations in cells derived from bovine papillomavirus lesions. 

As we have previously described in peripheral lymphocytes [15], the chromosome aberrations occur in significant increased levels in short-term lymphocyte cultures and in primary cell cultures, established from samples obtained from BPV affected animals. 

A very important point has to be discussed: the fact that it was not possible to detect virus DNA sequences in peripheral blood of affected or not affected animals. Despite this fact, the level of chromosome aberrations was verified higher in the affected animal samples. As it was not possible to identify virus DNA in blood either in control or affected animals, it could be discussed that the chromosome aberrations were not caused by virus action. However, the same types of aberrations were detected in cells derived from lesions, positive for virus presence. Furthermore, there was a significant difference between the chromosome aberration levels, comparing affected and not affected animals. 

In matter of fact, considering the similarity of the detected chromosome aberrations, the virus action on host chromatin was verified as effective either in blood cells or in cells derived from lesions. We could argue that the virus load in the peripheral blood of the affected animals was too low to be detected in conventional PCR. The same argument could be used for the not affected animals, leading to the need to compare affected to nonaffected animals and emphasizing the differences observed in the levels of chromosomal aberrations.

The virus action was verified rising numerical and structural chromosome aberrations. This fact indicates that the virus acts in different ways in its interaction with host chromatin. It was previously described the virus oncoprotein action on telomeres leading to centric fusion [28]. Besides, the virus acts on mitotic spindle, changing the chromosome set and rising aneuploidy [29]. 

Although BPV is described as nonintegrated in host cell, its action produces different types of chromosome alterations suggesting a large interaction with the chromatin and also with DNA repair mechanisms [30]. 

We have already described the presence of virus sequences in primary culture cells, in different passages [31], but now we demonstrate that these sequences are active leading to chromosome alterations. 

## 4. Conclusions

We compare for the first time the action of bovine papillomavirus on host cell chromatin in cultured lymphocytes and primary culture cells, describing the increase of chromosomal aberrations in both cell types. The primary cells cultures not only presented BPV DNA sequences but also keep these sequences throughout different passages. These data plus recent reports [18] emphasize possible active BPV infection in blood as well as in *in vitro *cultured cells. 

## Figures and Tables

**Table 1:** Chromosome aberration in peripheral blood cells and primary culture cells. C: control group; 1: cutaneous papillomatosis; 2: esophagus papillomas; 3: enzootic hematuria.

Group	Total cells	% Numerical aberrations (mean ± SE)	% Structural aberrations^§^ (mean ± SE)	% Cells with gaps (mean ± SE)
Blood				
C	126	7.86 ± 1.87	9.46 ± 0.97	3.93 ± 0.59
1	149	19.87 ± 4.59*	30.89 ± 7.71*	13.28 ± 4.50*
2	101	26.32 ± 5.53*	41.88 ± 2.25**	11.29 ± 3.77*
3	125	19.76 ± 4.69*	33.89 ± 9.18*	10.97 ± 1.92*

Primary culture cells				
C	124	14.43 ± 4.34	14.94 ± 1.85	7.16 ± 0.48
1	188	28.27 ± 3.14*	40.39 ± 1.40**	12.22 ± 4.48
2	164	46.39 ± 19.25^¤^	30.83 ± 0.37**	13.81 ± 0.67**
3	91	60.39 ± 0.55**	28.46 ± 1.35**	10.17 ± 3.92

*P≥ 0.05; **P≥ 0.01; ^¤^ *P*≥ 0.1; ^§^cells presenting only gaps were evaluated isolate.

**Table 2:** Frequencies of different aberrations detected in blood and primary culture cells of animals affected by cutaneous papillomas, esophagus papilloma, and enzootic hematuria.

Blood					
Group	CA	AT	AF	CB	CrB

C	0.88 ± 1.24	2.48 ± 1.46	2.33 ± 0.81	1.75 ± 2.48	1.60 ± 0.22
1	4.27 ± 2.20^¤^	3.86 ± 0.96	7.20 ± 5.27	2.31 ± 2.00	8.67 ± 3.03*
2	6.07 ± 1.28*	6.34 ± 5.67	14.74 ± 1.11*	1.16 ± 1.64	4.35 ± 3.71
3	1.65 ± 0.39	4.25 ± 2.14	6.86 ± 3.89	0.68 ± 0.97	6.31 ± 0.76**

	Ad/Del	EcrS	Aneu	Polip	Gaps

C	2.33 ± 0.81	0.72 ± 1.02	7.86 ± 1.19	0.72 ± 1.02	3.93 ± 0.59
1	15.02 ± 3.04**	2.85 ± 2.55	17.10 ± 3.57*	2.77 ± 1.02^¤^	17.81 ± 4.97*
2	26.28 ± 4.28**	11.55 ± 3.18*	23.14 ± 3.46*	3.19 ± 2.07	14.17 ± 2.98*
3	20.03 ± 7.02*	12.34 ± 3.86*	16.74 ± 6.24^¤^	3.02 ± 1.55	13.99 ± 3.46*

Primary culture					

Group	CA	AT	AF	CB	CrB

C	1.25 ± 1.77	3.01 ± 1.04	1.14 ± 1.61	0.63 ± 0.88	1.14 ± 1.61
1	5.61 ± 3.43^¤^	8.80 ± 3.50^¤^	5.79 ± 2.81^¤^	0.86 ± 0.75	5.59 ± 1.78*
2	5.16 ± 1.02*	4.74 ± 0.43^¤^	5.43 ± 1.74^¤^	0.42 ± 0.59	4.74 ± 0.42*
3	3.21 ± 1.01	7.94 ± 2.91^¤^	3.48 ± 2.15	2.23 ± 0.38^¤^	2.23 ± 0.38

	Ad/Del	EcrS	Aneu	Polip	Gaps

C	7.67 ± 2.01	5.28 ± 2.17	13.81 ± 3.46	0.63 ± 0.88	8.30 ± 1.12
1	32.59 ± 7.82*	9.95 ± 4.73	23.66 ± 6.13^¤^	3.57 ± 4.29	13.61 ± 5.73
2	26.36 ± 0.44**	3.63 ± 1.99	38.70 ± 23.83	7.68 ± 4.58^¤^	18.97 ± 1.69**
3	18.55 ± 1.49*	5.71 ± 2.53	52.72 ± 0.31*	6.42 ± 2.01*	13.11 ± 0.87*

*P > 0.05, **P > 0.01, and ^¤^ *P* ≥ 0.1.

CA: centromere association, AT: association of telomere, AF: acentric fragments, CB: chromosome break, CrB: chromatid break, Ad/Del: addition/deletion, EcrS: early chromatid separation, Aneu: aneuploidy, Polip: polyploidy, and gaps.
